# Black surfaces on ancient leather tefillin cases and straps from the Judean Desert: Macroscopic, microscopic and spectroscopic analyses

**DOI:** 10.1371/journal.pone.0303635

**Published:** 2024-06-13

**Authors:** Yonatan Adler, Ilit Cohen-Ofri, Yonah Maor, Theresa Emmerich Kamper, Iddo Pinkas

**Affiliations:** 1 Institute of Archaeology, Ariel University, Ariel, Israel; 2 Dead Sea Scrolls Conservation Laboratory, Israel Antiquities Authority, Jerusalem, Israel; 3 Analytical Laboratory, Israel Antiquities Authority, Jerusalem, Israel; 4 Department of Archaeology, University of Exeter, Exeter, United Kingdom; 5 Department of Chemical Research Support, Weizmann Institute of Science, Rehovot, Israel; Universidad de Sevilla, SPAIN

## Abstract

Tefillin are Jewish ritual artifacts consisting of leather cases, containing inscribed slips, which are affixed with leather straps to the body of the tefillin practitioner. According to current Jewish ritual law, the tefillin cases and straps are to be colored black. The present study examines seventeen ancient tefillin cases discovered among the Dead Sea Scrolls in caves in the Judean Desert. All seventeen cases display grain surfaces with a very dark, nearly black appearance. We start with a hypothesis that the cases were intentionally colored black in antiquity using either a carbon-based or iron-gall-based paint or dye. The aim of this study is to test this hypothesis by subjecting these tefillin cases to a battery of examinations to assess the presence of carbon and iron used as pigments, and of organic materials which may have been used as binding agents in a paint. The tests deployed are: (1) macroscopic and microscopic analyses; (2) multispectral imaging using infrared wavelengths; (3) Raman spectroscopy; (4) Fourier transform infrared spectroscopy (FTIR); and (5) scanning electron microscope (SEM) and energy dispersive X-ray (EDX) spectroscopy. The results of these tests found no traces of carbon-based or iron-gall-based pigments, nor of organic compounds which may have served as binders in a paint. These results suggest that our posited hypothesis is unlikely. Instead, results of the SEM examination suggest it more likely that the black color on the surfaces of the tefillin cases is the result of natural degradation of the leather through gelatinization. The Judean Desert tefillin likely represent tefillin practices prior to when the rabbinic prescription on blackening tefillin was widely practiced. Our study suggests that the kind of non-blackened tefillin which the later rabbis rejected in their own times may well have been quite common in earlier times.

## Introduction

The tefillin ritual is based on a literalist understanding of four very similar verses in the Pentateuch which instruct that something should “be as,” or should be “tied as,” a “sign” upon the arm, and concurrently that this same thing should “be as a reminder,” or “be as *ṭôṭāfōt*” (however this enigmatic word may have been understood), between the eyes (Exod 13:9, 16; Deut 6:8; 11:18). By the late second century BCE, these verses were being interpreted as instructing a highly complex ritual which came to be called in Hebrew: “tefillin” [1]. The basic components of the ritual entailed inscribing excerpts from the biblical books of Exodus and Deuteronomy onto thin slips made of animal skin, inserting these slips into two leather cases, and using leather straps to affix one of these cases to the arm and the other to the head of the tefillin practitioner. Rabbinic literature beginning in the early centuries of the first millennium CE records intricate rules surrounding the preparation of tefillin, among which is a regulation that the leather used for the tefillin cases and straps must be colored black (see below).

The earliest surviving tefillin cases and slips were uncovered in the middle of the twentieth century among the Dead Sea Scrolls, in several caves near Qumran and elsewhere in the Judean Desert [2]. These finds have been assigned depositional dates from the late first century CE until the early second century CE, and the inscribed slips have been variously dated paleographically from the middle of the second century BCE until the middle of the second century CE [3]. This assemblage includes at least 25 leather cases, most of which display a very dark, nearly black appearance on the surface of the leather. The present study seeks to determine if this black coloring is the result of intentional coloring of the leather in antiquity as rabbinic legislation prescribed.

### Jewish legal sources on black tefillin

The Leiden manuscript of the Talmud Yerushalmi provides the following statement attributed to an Amoraic (third—fourth century CE) sage: “R. Yose b. Bibai taught: [the requirement that] tefillin should be square and black is a law given to Moses at Sinai” [4]. The word “black” here is a gloss added in the upper margin of the thirteenth century manuscript, and so may not be original to the late antique text of the Yerushalmi. The Babylonian Talmud presents a similar statement, attributed to a different Amoraic sage, which singles out the tefillin *straps* for blackening: “Rabbi Isaac said: [the requirement that tefillin] straps be black is a law given to Moses at Sinai” [5, 6]. Subsequent rabbinic debates in the medieval period and into the modern era centered on whether the requirement that tefillin must be black applies only to the tefillin straps (as suggested by the Babylonian Talmud), or if it applies also to the tefillin cases whether by legal mandate (as suggested by the gloss in the Leiden manuscript of the Talmud Yerushalmi) or else by custom for reasons of aesthetics [7]. Regardless of rabbinic legal positions throughout the ages, by today it has become ubiquitous practice to paint all tefillin black both on the tefillin cases and on their straps.

### Assemblage of tefillin cases from the Judean Desert

The tefillin assemblage from the Judean Desert includes at least 25 leather cases. In three of these cases, fragments of leather straps were reported to have been discovered surviving in-situ. Photographs in the official archaeological reports on these artifacts are all low-quality reproductions in grayscale [8–13], with only one recently published exception which has been published in full color [14]. Scholars who published the official archaeological reports on these artifacts only rarely took note of the color on the exterior surfaces of the leather from which the tefillin cases or straps where made. Two tefillin cases were explicitly reported to have had a black surface [11, 15 p. 299–300]. Ronald Reed, a leather and parchment expert who analyzed one of these cases, asserted that: “the very intense region of stain on the grain side corresponds to a black dyestuff used for colouring this leather article” [15 p. 299]. Józef T. Milik, the scholar who published the majority of the Judean Desert tefillin cases, asserted that the standard procedure in preparing tefillin cases in antiquity was to begin with a piece of leather with a black surface (“à surface noire”; presumably he meant that the leather surface was purposefully colored so) from which a square was cut out and formed into a case [13 p. 34]. Yehuda Frankl, who analyzed the leather used in one of the Judean Desert tefillin cases, noted that “the leather seems to be undyed,” adding that its dark-brown color is a normal result of oxidation of the tanning material over time [16 p. 42]. Subsequent synthetic scholarly treatments of the Judean Desert tefillin assemblage [1, 2, 17] made no further reference at all to the color of the leathers used for the tefillin cases and straps.

**Fig 1:**
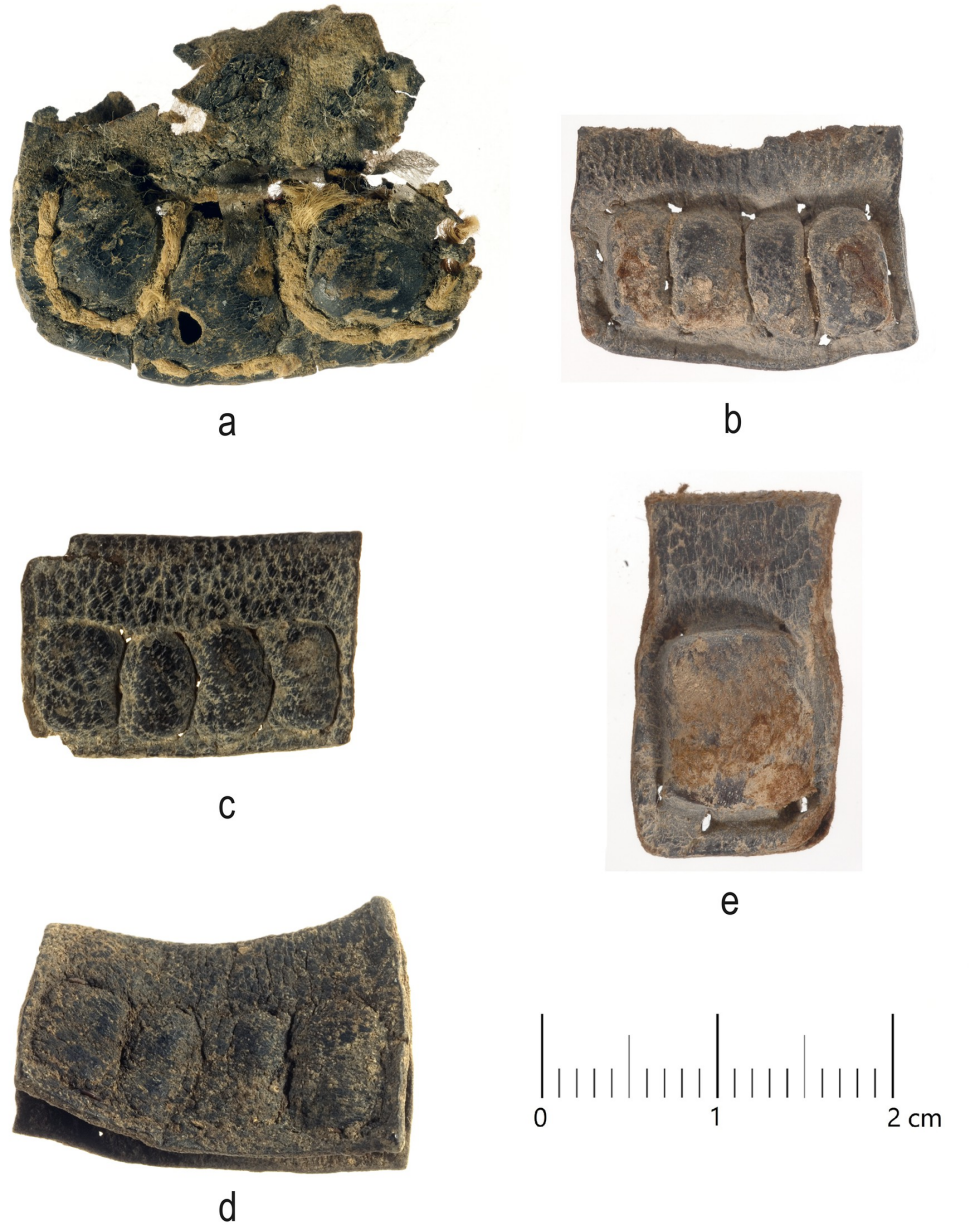
Examples of tefillin cases with very dark, nearly black grain surfaces: (a): tefillin case no. 4; (b) tefillin case no. 8; (c) tefillin case no. 9; (d) tefillin case no. 11; (e) tefillin case no. 16. Courtesy of the Israel Antiquities Authority (Photos: Clara Amit).

**Fig 2:**
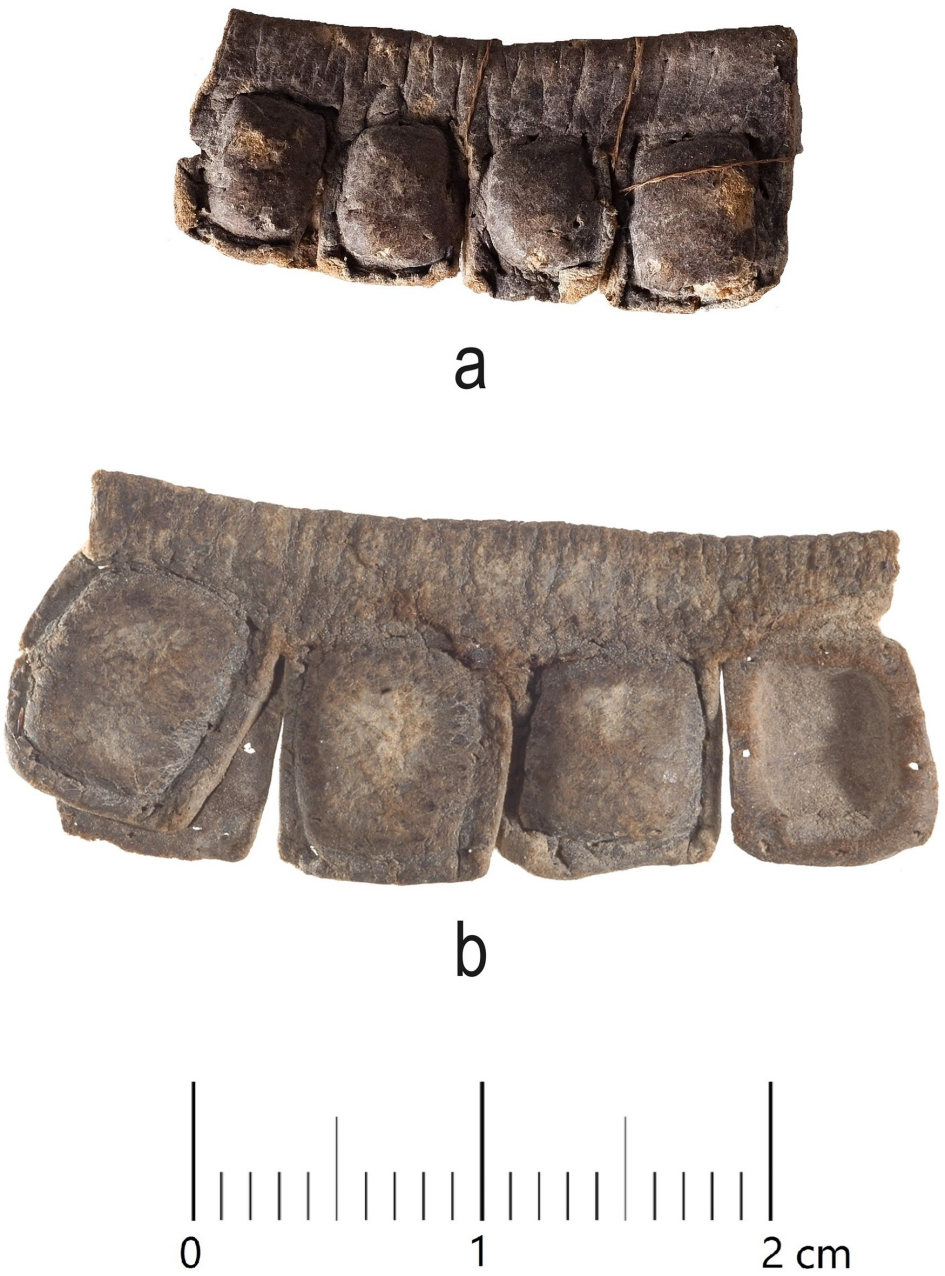
Examples of tefillin cases with brown grain surfaces: (a) tefillin case no. 18. Courtesy of the Israel Antiquities Authority (Photo: Ardon Bar Hama); (b) tefillin case no. 19. Courtesy of the Israel Antiquities Authority (Photo: Clara Amit).

Today, nineteen of the published tefillin cases are held at the Dead Sea Scrolls unit of the Israel Antiquities Authority in Jerusalem, and these were available to us for examination. A cursory visual inspection of these cases indicates that seventeen of these cases display a grain surface (papillary-dermal surface) which can best be described as either black or nearly black (Fig 1) (Table 1). Initial macroscopic examination does not reveal if this color is the result of intentional coloring in antiquity or if it is the result of natural processes which darkened the leather over the course of time. Two of the cases, along with an additional case held in the Shrine of the Book of the Israel Museum (the one mentioned above as having been analyzed by Yehuda Frankl), have grain surfaces which display a medium brown color (Table 2) (Fig 2).

**Table 1:** List of black-colored tefillin cases. The column “Multi-spectral imaging” indicates which of the tefillin cases has been photographed using multispectral imaging, including under both full-color and infrared wavelengths. All cases currently held at the Dead Sea Scrolls Conservation Laboratory—Israel Antiquities Authority, Jerusalem, Israel.

No.	Ascension no.	Plate no.	Publication	Multi-spectral imaging	Additional information
1	—	1343	[10 pl. 8:6]	✓	Found in Qumran Cave 8; strap fragment in-situ
2	—	1355	[10 pl. 8:5 (on right)]	✓	Found in Qumran Cave 8
3	—	1355	[10 pl. 8:5 (on left)]	✓	Found in Qumran Cave 8; strap fragment in-situ
4	352055	1008	[13 pl. 6:1,3]	✓	Found in Qumran Cave 4a
5	541018	1008	[13 pl. 6:2]	✓	Found in Qumran Cave 4a
6	538663	1008	[13 pl. 6:4]	✓	Purportedly from Qumran “Cave 4”
7	581855	1008	[13 pl. 6:6]	✓	Purportedly from Qumran “Cave 4”
8	581855, 581853	1008	[13 pl. 6:7]	✓	Purportedly from Qumran “Cave 4”
9	351294, 351296	1008	[13 pl. 6:8]	—	Purportedly from Qumran “Cave 4”
10	352053	1008	[13 pl. 6:10]	—	Purportedly from Qumran “Cave 4”
11	352056	1008	[13 pl. 6:11]	—	Purportedly from Qumran “Cave 4”
12	581852	1008	[13 pl. 6:12]	✓	Purportedly from Qumran “Cave 4”
13	—	1008	[13 pl. 6:13]	✓	Purportedly from Qumran “Cave 4”
14	—	1008	Not published	✓	Purportedly from Qumran “Cave 4”
15	—	100	[11 pl. 38:8]	✓	Found in Qumran Cave 5
16	351293	1008	[9 pl. 14:4]	—	Wadi Murabbaʿât Cave 1
17	—	2014–9017	[14 Figs 1–3]	✓	Naḥal Ṣeʾelim Cave 34

**Table 2:** List of brown-colored tefillin cases. The column “Multi-spectral imaging” indicates which of the tefillin cases has been photographed using multispectral imaging, including under both full-color and infrared wavelengths. Cases 18 and 19 currently held at the Dead Sea Scrolls Conservation Laboratory—Israel Antiquities Authority, Jerusalem, Israel. Case no. 20 currently kept at the Shrine of the Book—Israel Museum, Jerusalem, Israel.

No.	Ascension no.	Plate no./Item ID	Publication	Multi-spectral imaging	Additional information
18	581854	1008	[13 pl. 6:5]	✓	Purportedly from Qumran “Cave 4”
19	349867, 351295	1008	[13 pl. 6:9]	—	Purportedly from Qumran “Cave 4”
20	96.86.213	717710	[12 pl. 1–4]	—	Unknown; purportedly from a cave near Qumran

### Ancient literary sources on black pigments used in inks and leather dying

Ancient texts from the Mediterranean basin and dating to around the time of the Judean Desert tefillin cases describe two ways black colorants might be applied to parchment and leather: either as ink on writing supports or as dye on leathers used for footwear, clothing and the like [18, 19].

Three types of black ink are attested in the Greek and Latin sources: (1) “carbon ink” made with pigments based on soot or charcoal; (2) “iron-gall ink” made from mixing an iron sulphate with tannins such as oak gall; and (3) “mixed ink” made from carbon-based pigments mixed together with metal bearing minerals (copper, iron, lead) [18, 19]. In early rabbinic literature, the use of soot (Hebrew: *ʿāšānîm*, lit.: “smoke”) in the preparation of ink is attested [20], as is the use of what is likely iron sulphate (Hebrew: *qanqantôm* / *qanqantôs* / *qalqantôs* = Greek: *khálkanthon*) [21, 19 p. 182–183].

In all three types of ink described above, the inorganic pigments (carbon and/or metals) are mixed with an organic binding agent, which acts like a glue, and then (immediately before use) suspended in water or another aqueous solution to create a liquid ink suitable for writing [19]. Ancient textual sources refer to binding agents made from gum arabic (a sticky exudate from the stem and branches of acacia trees), bees’ honey, oil, and animal bone glue [19, 22]. The use of gum arabic (Hebrew: *qômôs* / *qômāʾ* = Syriac = *qômāʾ* = Greek: *kómmi*) as a binder in ink is attested in early rabbinic sources [21], as are other kinds of resins and oils [20].

Ancient authors writing in Latin and Greek describe a dye used for coloring leather black (Latin: *atramentum sutorium*; Greek: *khálkanthon*) which is based on the same chemistry as iron-gall ink mentioned above [19]. One example is Pliny the Elder (writing circa 77–79 CE), who described various ways that the dye was prepared [23]. The organic binder that is used in ink suspensions is superfluous here, as the dye is meant to be absorbed into the leather rather than sit on the surface [18 p. 92].

### Chemical characterizations of black inks

Studies in recent years have made use of several analytical methods to chemically characterize black inks. For example, X-ray fluorescence (XRF), Fourier-transform infrared (FTIR) spectroscopy, and scanning electron microscopy (SEM) with energy dispersive X-ray (EDX) spectroscopy have all been deployed to characterize black inks used on fragments of the Dead Sea Scrolls [24, 25]. Optical microscopy, near infrared spectroscopy, XRF, SEM-EDX spectroscopy, fiber optics reflectance spectroscopy (FORS), and molecular investigation by micro-Raman spectroscopy have been used to characterize black inks used on Egyptian papyri [26]. These studies have demonstrated the presence of amorphous carbon (i.e., a noncrystalline, solid allotropic form of carbon) which served as the black pigment in the inks. Similar methods have been used to characterize black inks from late antiquity (beginning in the fourth century CE) and onwards which used iron-gall as the basis for the black pigment [27–30]. And finally, FTIR has been shown to be useful for identifying organic binders in inks, such as gum arabic [31, 32].

### Hypothesis to be tested in the present study

Considering later rabbinic legislation and practice, and considering the known ancient methods for applying black color to skin artifacts, in the present study we propose the hypothesis that the tefillin cases from the Judean Desert which display black surfaces were artificially colored black in antiquity using either carbon-based paint or dye, or else an iron-gall-based paint or dye. The aim of our study is to test this hypothesis by subjecting these tefillin cases to a battery of examinations to test for the presence of carbon black and iron, as well as to test for the presence organic materials extraneous to the leather which may have been used as binding agents in a paint.

## Materials and methods

**Fig 3:**
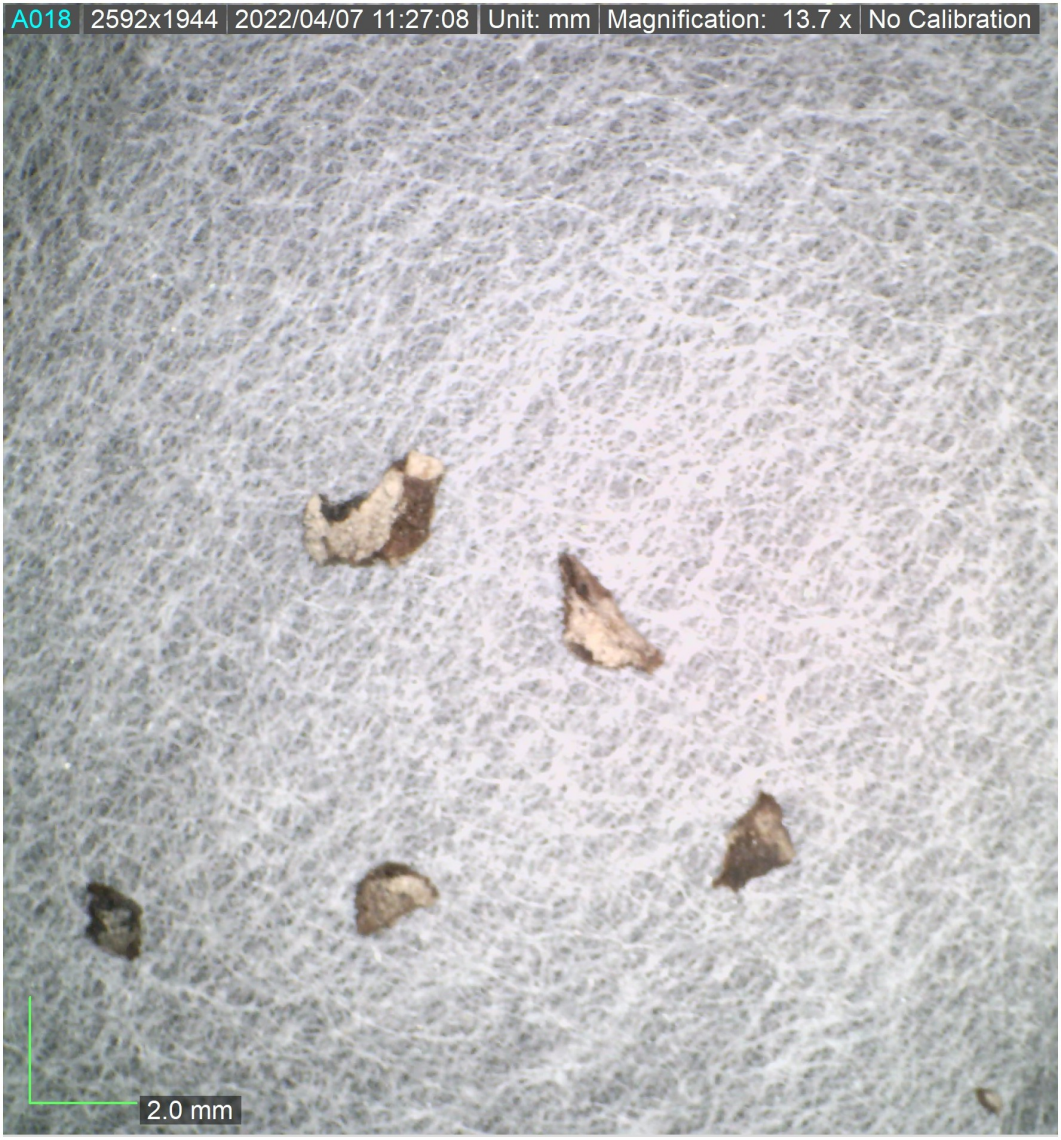
Fragments from tefillin case no. 1. Six fragments of leather which had broken off from tefillin case no. 1, collected and subjected to further microscopic, Raman, FTIR, and SEM-EDX analyses. (Photo: Ilit Cohen-Ofri).

**Fig 4:**
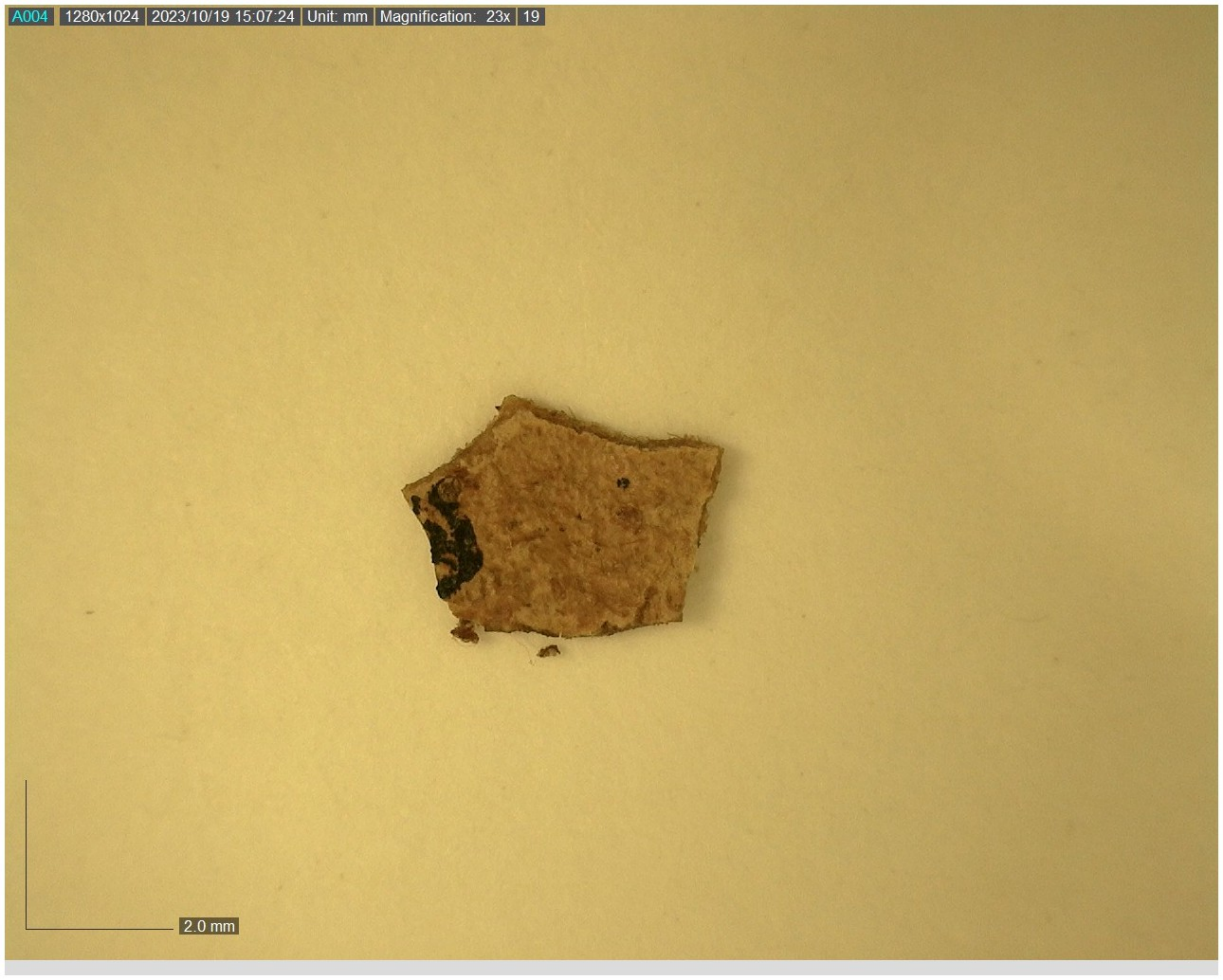
Parchment fragment with ink from Qumran Cave 11 (Box 1010). Image taken with Dino-Lite microscope (Photo: Yuliya Shmidov).

All seventeen tefillin cases with black-colored surfaces were subjected to macroscopic (visual inspection) and microscopic (stereoscope) analyses. Multispectral imaging was taken of thirteen of these tefillin cases (see Table 1). Six small fragments of leather that in the past had unintentionally broken off from tefillin case no. 1 (Fig 3) were sampled in order to perform further microscopic, Raman, FTIR, and SEM-EDX analyses. In addition, a fragment of parchment with a small ink spot from Qumran Cave 11 (Museum inventory Box 1010) (Fig 4) was subjected to Raman analysis for ink and parchment comparison. Considerations of artifact conservation disallowed further sampling from other, intact tefillin cases.

### Color characterization

Quantitative color characterization was conducted on four tefillin cases (nos. 7, 8 and 12), and this analysis was used as the benchmark for visually characterizing the remainder of the exemplars. A full-color image of the cases was taken with a Eureka Vision LED system manufactured by MegaVision, Santa Barbara, CA, USA. The system is equipped with a 39-megapixel Kodak CCD E6 monochrome sensor array, and four Eureka Light LED illumination panels. The camera produces images quantized to a dynamic range of 12 bits per channel. The spectral component is provided by fixed wavelength light emitting diodes (LEDs) that emit in narrow spectral bands. The image is taken in twenty-eight exposures under twelve different wavelengths—seven in the visible light spectrum (445 nm–656 nm) and five in the near-infrared (NIR) spectrum (706 nm–924 nm)—creating a folder of fifty-six monochromatic exposures per fragment. The system then generates a full-color image by combining the visible light wavelengths. Imaging took place in the laboratory of the Dead Sea Scrolls unit of the Israel Antiquities Authority, in Jerusalem. Colors were then compared on this image according to the L*a*b* color space, where L* represents lightness with 0 as total black and 100 as white. The a* axis ranges from red to green with positive values being redder and more negative values are greener. The b* axis ranges from yellow to blue with yellow having positive values and blue negative values.

**Fig 5:**
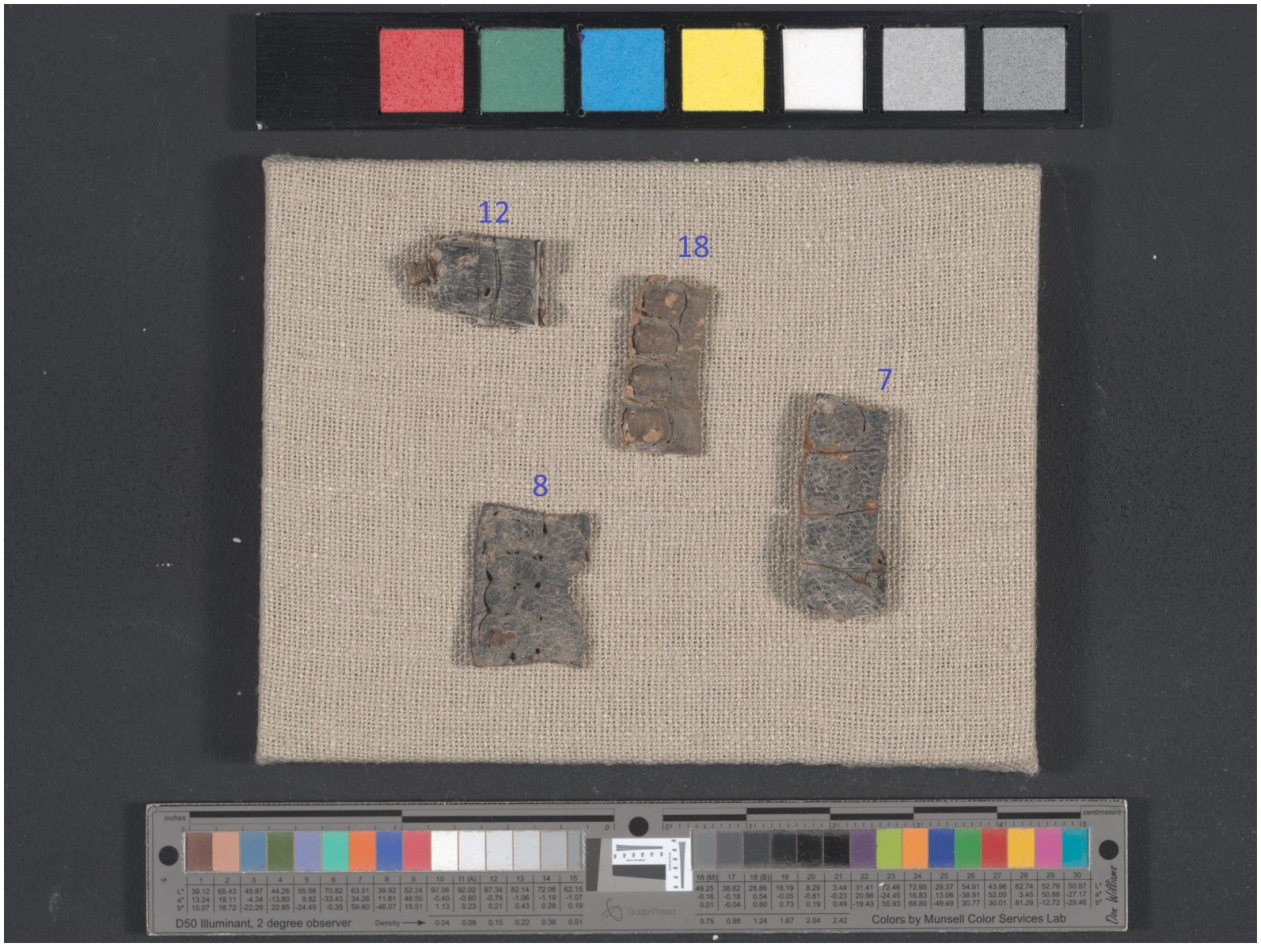
Full-color image of tefillin cases nos. 7, 8, 12, and 18. Photographed using the multi-spectral system for color comparisons. Courtesy of the Leon Levy Dead Sea Scrolls Digital Library; Israel Antiquities Authority (Photo: Shai Halevi).

Fig 5 shows the full-color image of the four cases, three of which were described as “black” (nos. 7, 8 and 12) and one as “brown” (no. 18). On each case, five spots from various areas were measured, choosing dark spots where the grain layer appears intact, avoiding areas of exposed reticular dermis. Each measurement area is approximately 1 mm^2^. The measurements from each case were averaged and the standard deviation calculated (the results are summarized in Table 3). The brown case is slightly lighter than the three black, as seen by the lower L* value. However, the differences in lightness are small relative to the natural variation across the cases; the more important distinction is that “black” cases are colorless, having a* and b* values very close to zero, while brown cases have positive values in both, indicating slightly yellow and red color components. These results illustrate quantitively what is meant by “brown” or “black” throughout this study.

**Table 3:** L*a*b* color space values of cases in Fig 5. n = 5, errors are the standard deviation.

Apparent color	Tefillin case no.	Average L*	Average a*	Average b*
Black	7	38.6 ± 0.9	0.4 ± 0.5	0.6 ± 0.9
Black	8	38.0 ± 1.2	0.4 ± 0.5	1.0 ± 0.0
Black	12	35.8 ± 5.0	0.4 ± 0.5	1.0 ± 0.7
Brown	18	41.2 ± 1.8	3.8 ± 0.8	6.4 ± 1.1

### Microscopic analysis

Macroscopic and microscopic analyses were undertaken for an initial determination of the location, distribution, and behavior of the darker color on the tefillin cases. Paint applied to the leather could be expected to appear localized as a coating on the surface, and typically would not be expected to penetrate deeply into the structure of the leather. Dye applied as a stain would be expected to present as a color absorbed into the surface of the leather.

**Fig 6:**
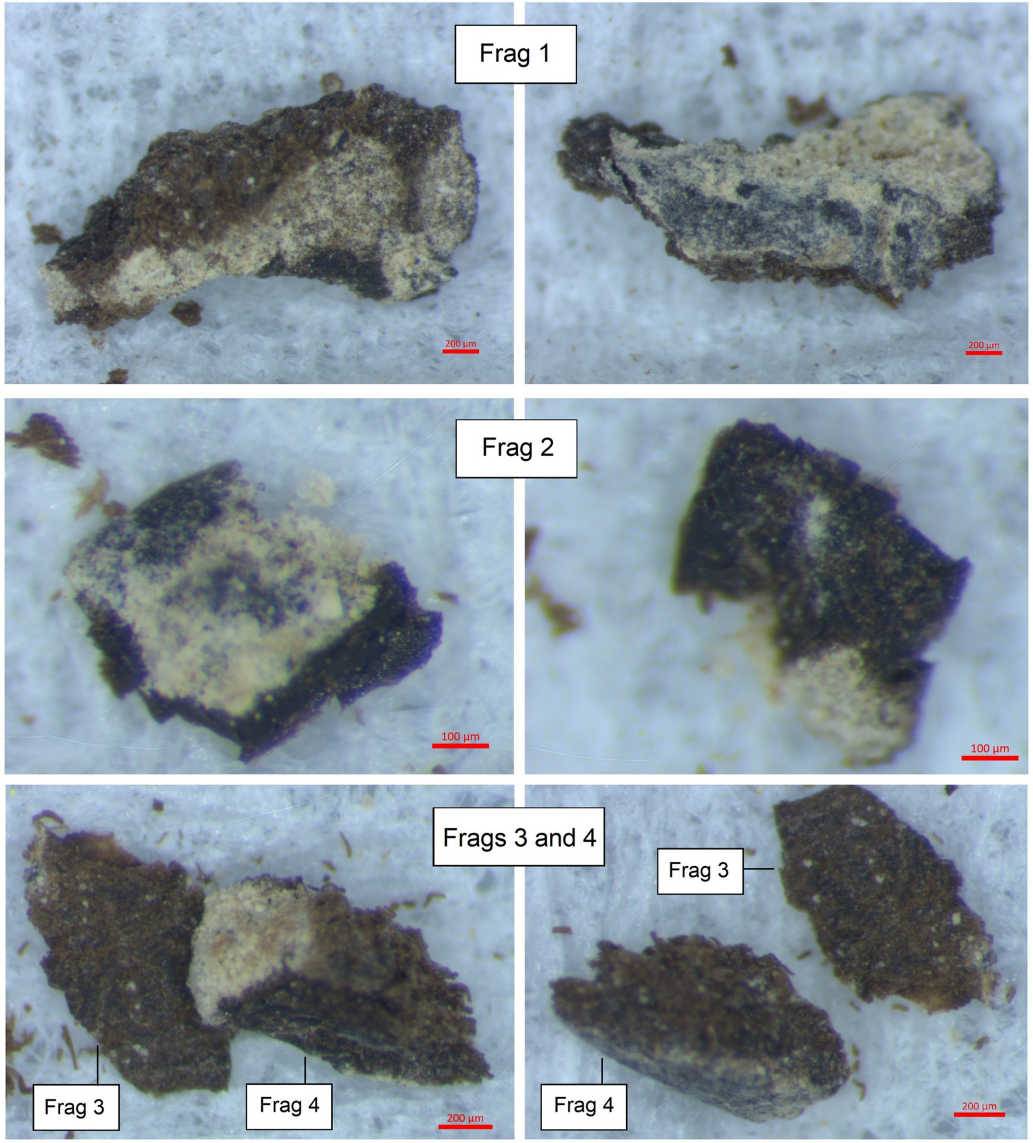
Photomicrographs of fragments from tefillin case no. 1. Frags 1, 2, 3, 4 (Photo: Yonah Maor).

**Fig 7:**
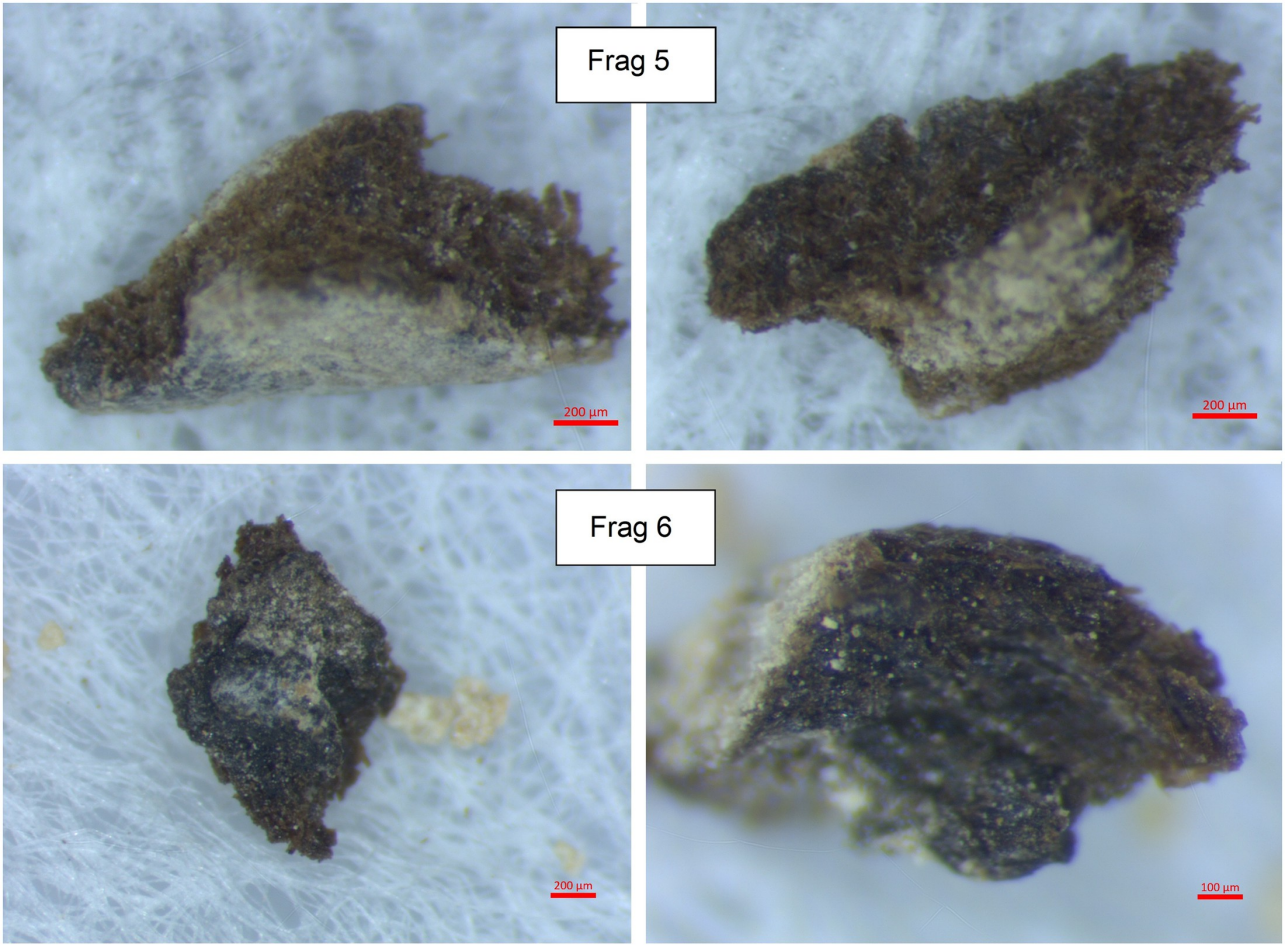
Photomicrographs of fragments from tefillin case no. 1. Frags 5, 6 (Photo: Yonah Maor).

All of the seventeen tefillin cases with blackened surfaces were subjected to microscopic analysis using an Amscope ZTX-3E stereo microscope, with recording of microscopic images undertaken using an Amscope 5MP FMA050 digital camera system. All cases were also examined using a Dino-Lite AM7915MZT 5MP microscope, with recording of microscopic images undertaken using DinoCapture for Windows and DinoXcope for MacOS. The six small fragments of leather that had broken off from tefillin case no. 1 were analysed using a Zeiss Stereo Discovery V12 microscope, and a 5 megapixel Axiocam 105 color camera was used to take photomicrographs of these fragments prior to further additional analyses. The fragments were assigned as Frags 1–6 (Figs 6 and 7).

### Multispectral imaging analyses

**Fig 8:**
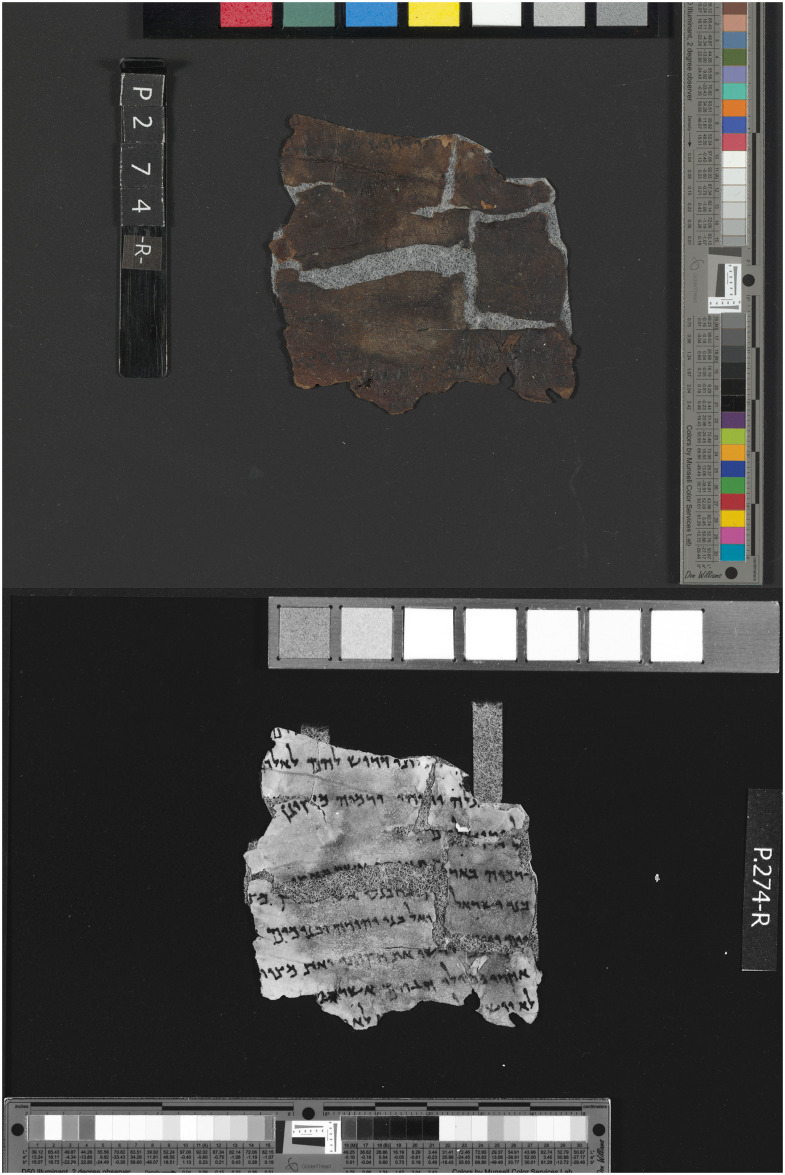
Example of near infrared image of Dead Sea Scroll with carbon ink. 4QApocryphon of Jeremiah^c^ (4Q385b), Frag. 16 II; top: full spectrum color image; bottom: near infrared (924 nm) image. Courtesy of the Leon Levy Dead Sea Scrolls Digital Library; Israel Antiquities Authority (Photos: Shai Halevi).

Multispectral imaging analyses test for the presence of pigments containing amorphous carbon. The difference in reflectance between leather and amorphous carbon pigments increases as the wavelength moves toward near infrared (700–924 nm), at which point the contrast between reflective leather and absorbent carbon is stark. (This contrast obtains even on naturally darkened skins, as in some of the Dead Sea Scrolls; see, e.g., Fig 8).

Imaging of thirteen tefillin cases (see Table 1) was conducted using the MegaVision Eureka Vision LED system described above, and images taken under near infrared light (924 nm) were compared with full-color images.

### Raman spectroscopy

Raman spectroscopy is used to reveal the presence of amorphous carbon molecules as well as the presence of molecules of various other minerals and certain organic matter that can be used as pigments in a black paint or dye, e.g., iron-gall.

The tefillin case no. 1 fragments and parchment fragment from Cave 11 (Box 1010) were placed on a microscope-bearing glass and dark and brighter areas were photographed and measured. The Raman measurements were collected using a Horiba LabRAM HR Evolution^®^ micro-Raman instrument with four lasers, for this study a near infrared laser (785 nm). Images were acquired at 10×, 50×, and 100× magnifications, and spectra were acquired using a 600 gr/mm grating. The spectra were acquired using roughly 1.6mW of laser power with the 50× objective (spot size about 1 μm). The acquisition lasted 10 seconds and was averaged 6 times. The spectra were background corrected using polynomial subtraction and some were smoothed lightly using a 3^rd^ order Savitzky-Golay filter with 7 neighbors as is commonly done. The measurements were collected at the Optical Spectroscopy and Microscopy Lab in the Department of Chemical Research Support at the Weizmann Institute of Science in Rehovot, Israel.

### FTIR-ATR spectroscopy

Fourier transform infrared spectroscopy (FTIR) is used with an attenuated total reflectance (ATR) attachment to identify minerals and molecules of organic compounds related to black paints and dyes that could not be identified using Raman. This test is destructive, as it involves pressing samples in order to obtain a spectrum with reasonable signal to noise.

Two samples taken from the fragments which had broken off from tefillin case no. 1 were analyzed:

**Sample 1**: Taken from Fragment 2 (Fig 6); chosen as an example of leather which displays a black colored surface.
**Sample 2**: Taken from Fragment 3 (Fig 6); chosen as an example of leather which displays a lighter, brown colored surface.

Both samples were pressed against the diamond in the ATR for analysis. Infrared spectra were collected using a Nicolet iS5 FTIR spectrometer with an iD7 diamond ATR attachment. The Spectra shown are from 32 scans collected at 4 cm^−1^ resolution in the 525–4000 cm^−1^ range.

### SEM and EDX spectroscopy

Scanning electron microscope (SEM) and energy dispersive X-ray (EDX) spectroscopy is used to gain a high magnification imaging of the analyzed area and to identify various elements which make up the minerals and molecules of organic compounds related to black paints and dyes which could not be identified using the above two spectroscopy methods. Measuring the relative amount of elements in the sample is done by means of EDX whereas SEM is used to obtain a high magnification image of the analyzed material.

Two fragments from tefillin case no. 1 were chosen for SEM analysis and EDX spectroscopy: Frags 1, 2 (Fig 6). Fragment 1 was chosen because it was on the larger size and contained brown, black, and mineral coated areas—all on the same fragment. Fragment 2 was chosen as a mostly black example, though sections appear lighter due to mineral coating.

All fragments were placed on a carbon tape sited on a stub and sprayed with compressed air to prevent the sample from being detached into the device. The stub was placed on a dedicated surface inserted into the vacuum chamber. Analysis was done with a low vacuum of 60 Pa to avoid damaging the fragments and to minimize charging effects. Different areas of the sample were photographed, and the chemical elements were mapped by means of the EDX detector. EDX quantification was based on the XPP algorithm [33] which is an extension of Phi-Rho-Z method and is calibrated on a copper-aluminum standard. The accelerating voltage was 15 keV for EDX or 5 keV for images; additional specific settings such as working distance and magnification are listed on the images. The measurement was carried out with a Thermo Scientific TM Phenom XL desktop SEM with an EDX detector.

## Results

### Macroscopic and microscopic analyses

Macroscopically, all seventeen cases under examination display a grain surface with a very dark, nearly black color. The edges of the leather (in section) are not the same color or texture as the grain surface and no dark coloration is seen on the stitching material where this has survived. There is some variability between the tefillin cases with regard to the texture of the grain surfaces of the leather: whereas on most cases the surface is moderately reflective and moderately smooth to very smooth, on some cases (tefillin cases nos. 7, 8, and 9) the surface is textured to rough.

**Fig 9:**
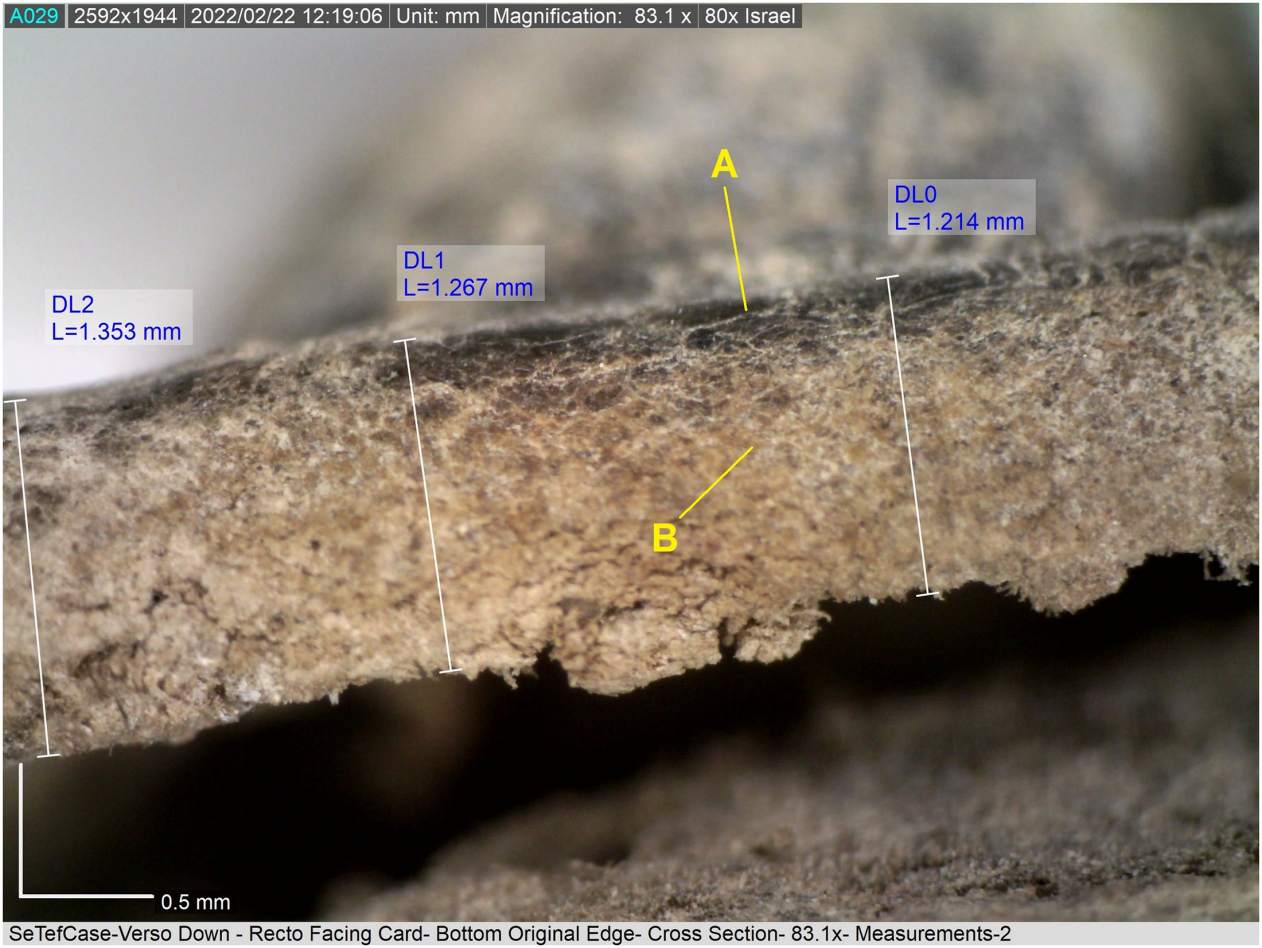
Tefillin case no. 17, cross section of leather. (A) Black-colored grain surface; (B) lighter-colored layer beneath the grain surface (reticular dermis). Under 83.1× magnification, Dino-Lite microscope (Photo: Theresa Emmerich Kamper).

**Fig 10:**
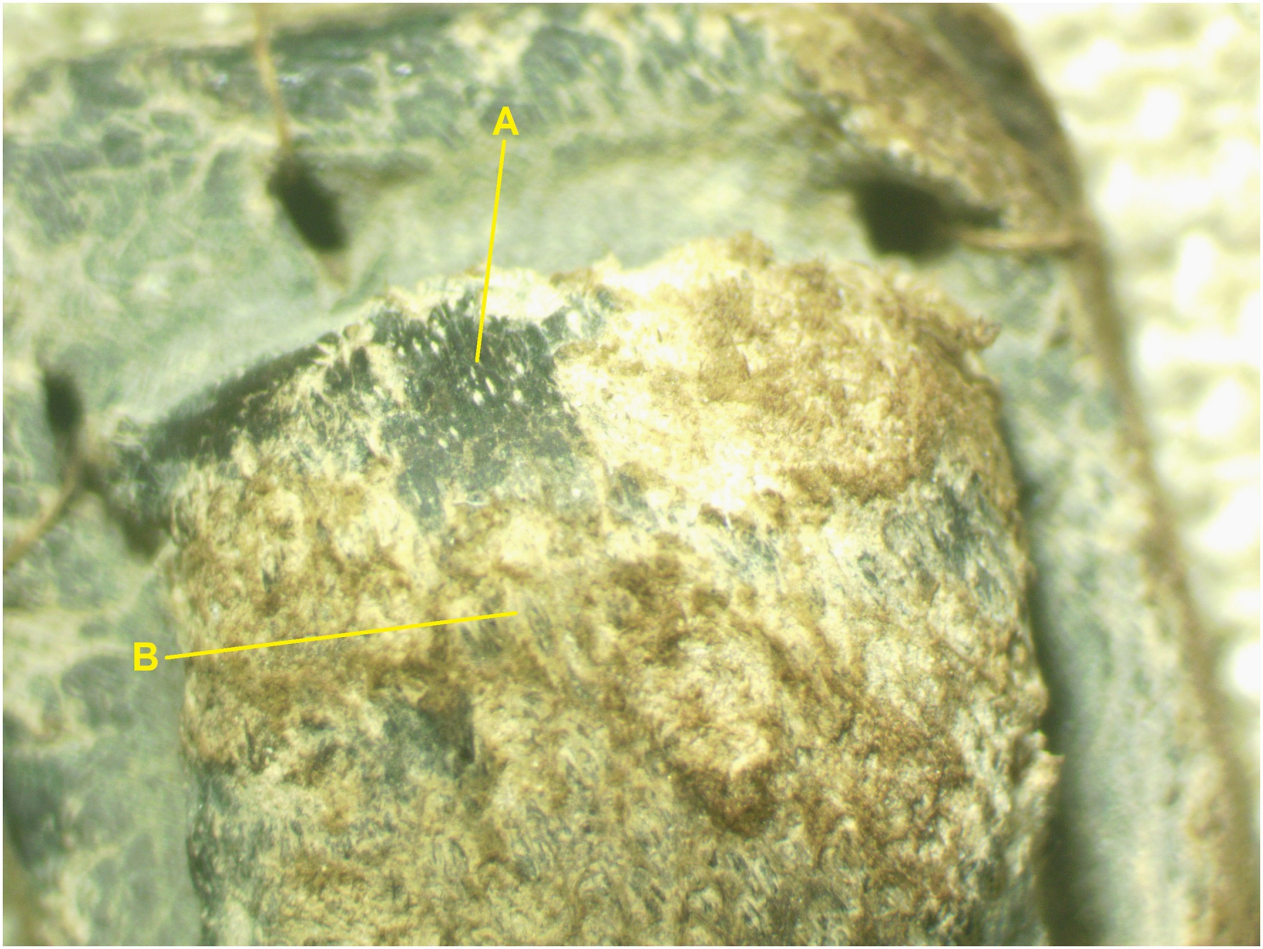
Tefillin case no. 11, surface. (A) Black-colored grain surface; (B) lighter-colored layer beneath the grain surface (reticular dermis). Under 10× magnification, Amscope microscope (Photo: Theresa Emmerich Kamper).

Under the microscope, it is observed that the dark coloration almost invariably does not penetrate past the papillary dermal layer into the reticular dermis. This is evident on cross sections of the leather (Fig 9) and on surfaces in areas where the grain surface has degraded and only the lower, reticular-dermal layer has survived (Fig 10). An exception to this is tefillin case no. 1, where dark coloration can be seen extending into the dermis in sections created by some, but not all of the insect holes on this artifact.

Microscopic examinations of the surfaces of several fragments which broke off from tefillin case 1 (Figs 6 and 7), reveal surfaces with a gradual transition across the surface from brown to darker brown or black. These images show that the dark color is fully integrated with the leather fibers and is not a coating sitting on the surface. On some fragments, for example Fragment 1 and Fragment 6, the darker areas of the surface look *lower* than the brown areas of the surface, as though the leather had contracted there. If the dark color had been intentionally applied to the entire surface but had degraded or flaked off in some spots, the dark area would have been higher than the brown rather than vice versa. In all cases the light grey-yellow color is simply a surface coating of minerals.

### Multispectral imaging analyses

**Fig 11:**
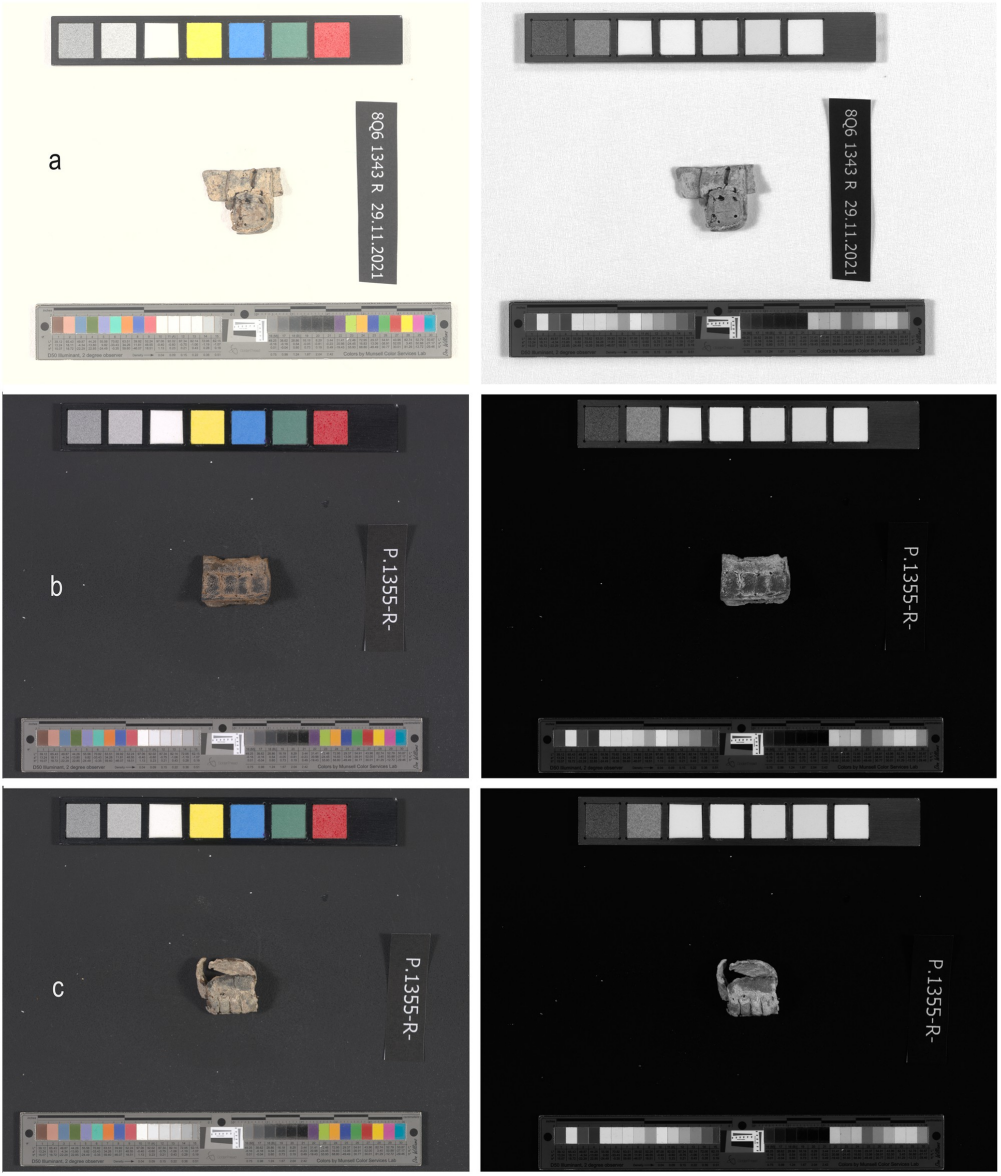
Examples of tefillin cases under full color light (left) and near infrared (924 nm) light: No anomalous absorbance of near infrared light on areas which appear black under full color light. (a) Tefillin case no. 1; (b) Tefillin case no. 2; (c) Tefillin case no. 3. Courtesy of the Leon Levy Dead Sea Scrolls Digital Library; Israel Antiquities Authority (Photo: Shai Halevi).

Multispectral imaging analyses of thirteen tefillin cases revealed that areas which appeared very dark to nearly black under full color light showed no anomalous absorbance of near infrared light (924 nm) (Fig 11), suggesting that amorphous carbon paint is not present on any of the tefillin cases analyzed.

### Raman spectroscopy

The two typical peaks of amorphous carbon are found at ~1590 cm^-1^, noted as the G peak (for “Graphite”) and an additional peak at ~1390 cm^-1^, called the D peak (for “Disorder”). The G peak is typical of the basic molecular structure of graphite while the D peak is characteristic of disordered graphite—both of which are present in materials such as soot, charcoal and bone black.

**Fig 12:**
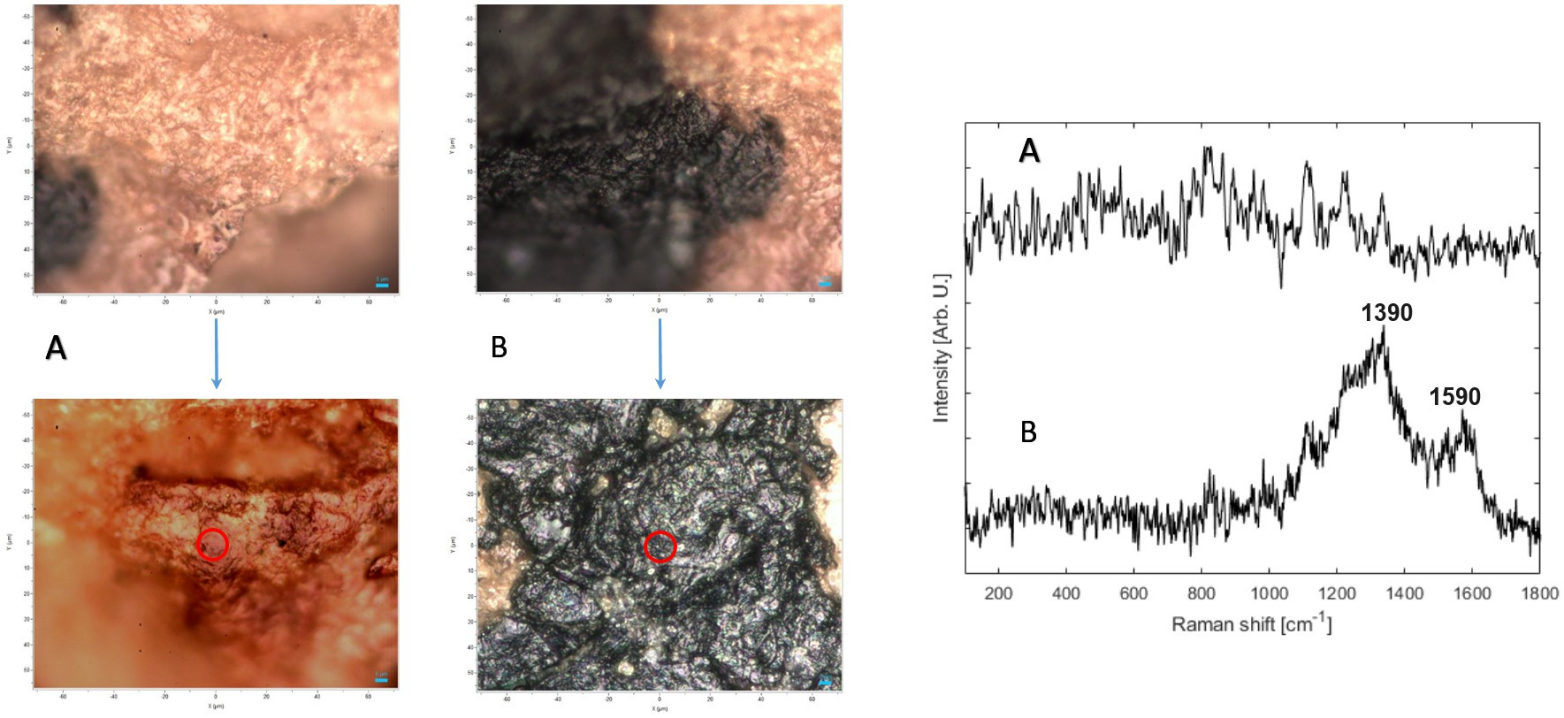
Qumran Cave 11 parchment fragment: Images taken with the Raman microscope and Raman spectra. Left (upper and lower images) and center (upper and lower images): images taken with the Raman microscope on the analyzed areas (red circles) where A is the light color analysis area (parchment) and B is the ink analysis location. Right: the Raman Spectra of analysis areas A and B. (Photos: Iddo Pinkas).

Analyses conducted on the parchment fragment from Qumran Cave 11 (Box 1010) with a small spot of ink residue (Fig 4) revealed that where no ink is apparent, the spectrum is associated with the parchment while analysis of the ink revealed that the two typical peaks of amorphous carbon 1590 and 1390 cm^-1^ were dominating the spectrum with small elements from the parchment below (Fig 12).

**Fig 13:**
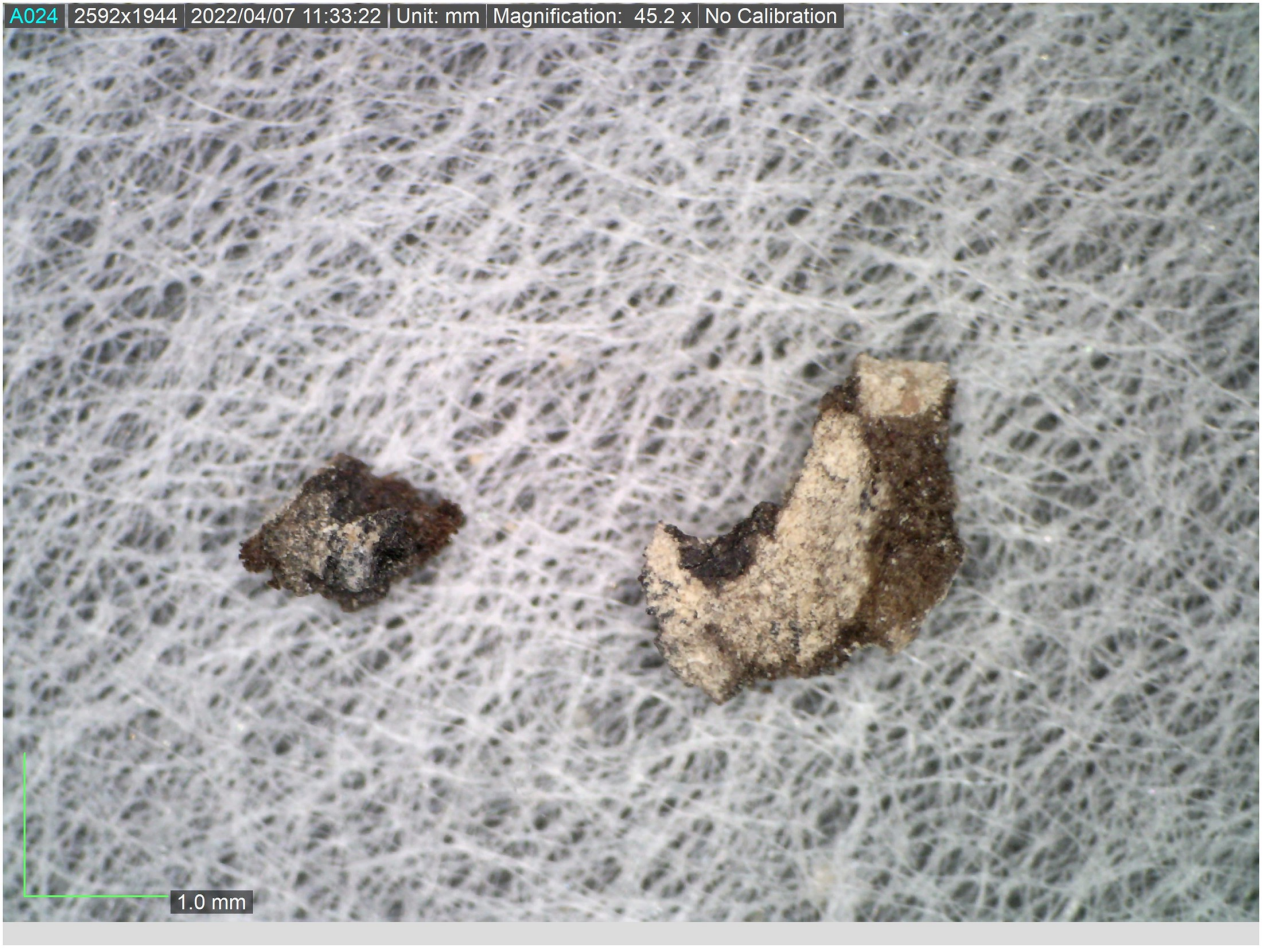
Two fragments broken off from tefillin case no. 1 chosen for Raman analysis. Images taken with Dino-Lite microscope (Photo: Ilit Cohen Ofri).

**Fig 14:**
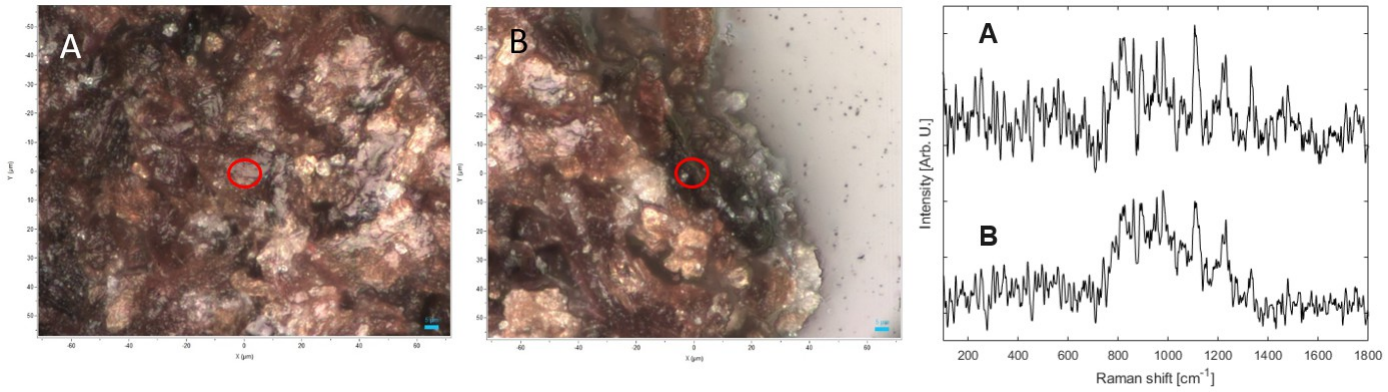
Two fragments broken off from tefillin case no. 1: Images taken with the Raman microscope and Raman spectra. Left and center: images taken with the Raman microscope on the analyzed areas (red circles) where A is the light color analysis area and B is the black color analysis location. Right: the Raman Spectra of analysis areas A and B. (Photos: Iddo Pinkas).

Tefillin fragments that were broken off from tefillin case no. 1 (Fig 13) were similarly analyzed. In both fragments, black areas and those that are lighter in color revealed peaks that associate with the Qumran Cave 11 parchment (Fig 14). We were not able to identify the molecular origin of these peaks, but we believe that they may be due to degraded organic materials such as proteins, polysaccharides, or fatty acids. The amorphous carbon peaks—namely peaks located at 1590 and 1390 cm^-1^—were not detected, nor were any minerals that can be attributed to pigments used for black paint.

Iron sulfate or complex of iron with tannins (the main ingredient in iron-gall ink) was not found in the samples. Its indicative peaks—strong peaks at 1575, 1470, 1425 cm^-1^ and 1327 cm^-1^, as well as a strong peak at 600 cm^-1^, and a weak peak at 400 cm^-1^ [34, 35]—were not detected.

### FTIR spectroscopy

**Fig 15:**
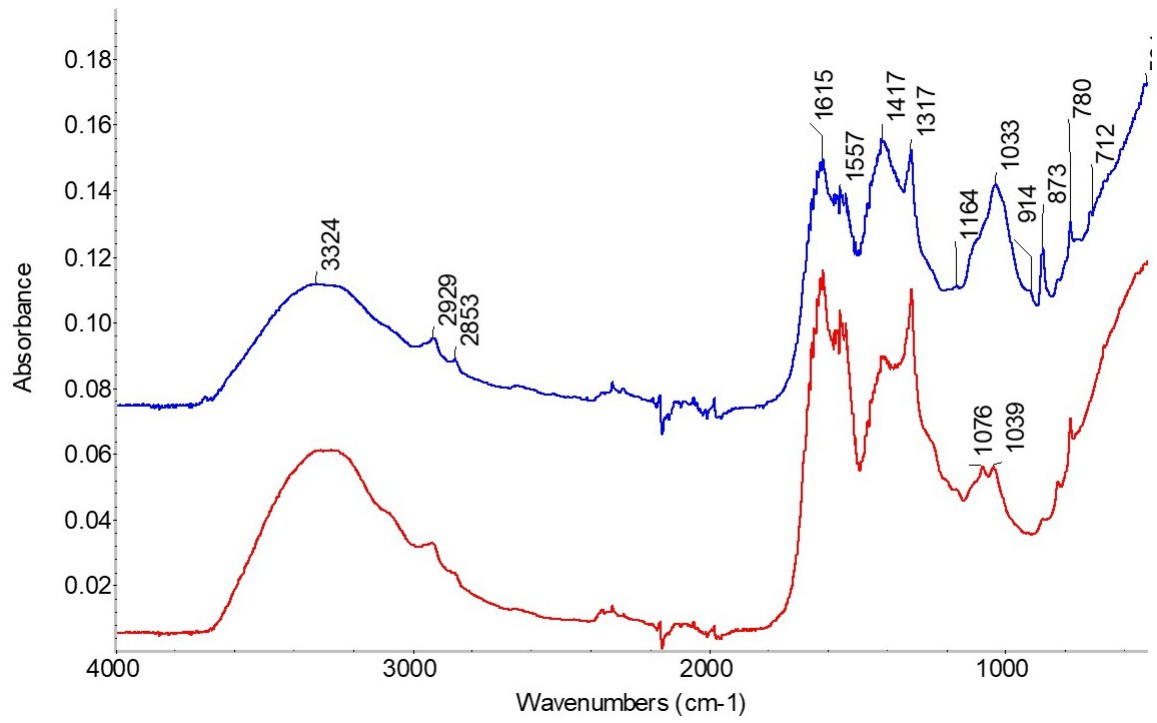
FTIR-ATR spectra of dark fragment (Sample 1—Blue) and lighter fragment (Sample 2—Red). Curve shifted for clarity.

Fig 15 shows the comparison of the two spectra resulting from our analyses of Sample 1 and Sample 2 using FTIR spectroscopy. The main differences between the two are the minerals attached to the black Sample 1. Peaks at 712, 873 and 1417 cm^-1^ are due to calcite, while peaks at 914, 1033 and 1164 cm^-1^ are silicate minerals—mainly clay and quartz. The peaks at 780, 1317 and 1615 cm^-1^ are due to calcium oxalate, which is a common precipitate in this region of the Judean Desert [36]. The main component of leather is collagen, represented by the amide I and II peaks at 1557 and 1615 cm^-1^ in both these samples. The amide I at 1615 cm^-1^ overlaps with the main peak of calcium oxalate; this is unfortunate as the height of that peak could otherwise have been used to assess the extent of collagen hydrolysis [37, 38]. Gelatinization, another form of protein degradation, is typically seen by a shift in the location of the amide II peak; however, due to the small sample size, these spectra have too much noise and accordingly we cannot assess the presence of gelatinization in these samples on this basis alone. The last component of chemical degradation pathways is oxidation. If oxidation were present, it would appear as a shoulder on the amide I peak, around 1730 cm^-1^; that part of the spectrum is clear and there is no sign of oxidation in either sample. Therefore, oxidation can be ruled out as a significant cause of the dark colors on these samples.

The peaks at 2853 and 2929 cm^-1^ are due to CH_2_ and CH_3_ bonds, which exist in most organic materials but are more prevalent in fats and oils. The peaks are quite low in both these when compared to scroll fragments from the Judean Desert caves [39], artifacts which presumably experienced similar degradation; we may conclude, therefore, that this tefillin was probably not treated with high quantities of fat or oil.

In Sample 1, no peaks can conclusively be attributed to tannins, which were most likely used to convert the animal skin to leather. Tannins typically have different patterns of peaks in the range 1000–1500 cm^-1^ depending on their source [40], but these were most likely hidden by overlapping mineral peaks that have stronger absorption. In Sample 2, which does not have as heavy a mineral coating, peaks at 1039 and 1076 cm^-1^ are revealed, as well as a more distinct shoulder around 1250 cm^-1^. These do not perfectly fit any of the references reported by Bicchieri and her co-authors, or the tannins observed on lightly tanned parchment of the Dead Sea Scrolls [39], but it is possible that a different source of tannins was used for leather tanning in this case.

### SEM and EDX spectroscopy

**Fig 16:**
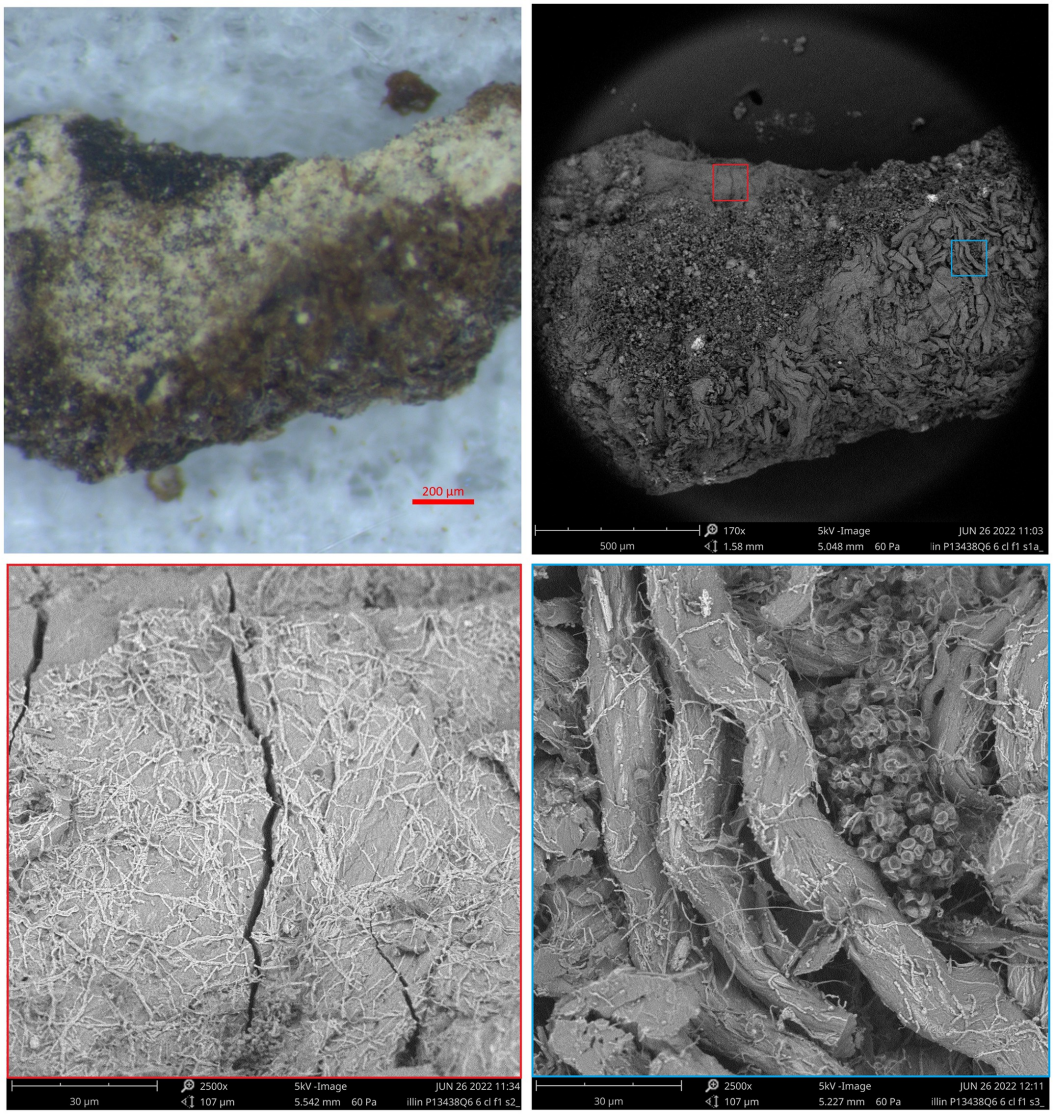
Fragment 1: Photomicrograph and SEM-BSD images. Upper left: Photomicrograph showing the same area as shown in color photomicrograph Fig 6. Upper right: SEM-BSD image with red square marking black area and blue square marking brown area. Lower left: SEM-BSD image of red square magnified 2500×. Lower right: SEM-BSD image of blue square magnified 2500×. (Photos: Yonah Maor).

In SEM back-scatter detector (BSD) images, the greyscale color is related to the chemistry as well as the topography of the object, with lighter grey relating to heavier elements rather than the visible color. Fig 16 shows the SEM images from Fragment 1 compared to the color photomicrograph. Thanks to the depth of field in the SEM image, the fact that the dark area is lower than the brown area is confirmed. Zooming in on the dark area (red square), we see that the fibrous structure is lost entirely, indicating that the leather is fully gelatinized. Some small micro-fibrils appear on the surface in light grey; these are calcified. The gelatinized area is more brittle, without the flexibility afforded by the original fiber structure, and cracks are visible all over this section. This is consistent with natural degradation which can be caused by exposure to water and heat and/or the passage of significant amounts of time. The dark color is associated with compaction of the translucent collagen fibers and formation of melanoidin type pigments as part of the degradation process [41]. Zooming in on the brown area (blue square) shows the leather’s fibrous structure is preserved. The area shown also includes some microbial spores, each with deep indentations suggesting that they are dead and have lost their internal content [42].

These two areas on Fragment 1 were also analyzed with SEM-EDX to determine their elemental composition. Due to the uneven surface of the sample, the quantification is not precise; however, the results reported in Table 4 are used to check the presence of elements that could be related to color, and qualitatively compare the two areas. The table shows that the brown and black areas have the same order of magnitude across all the elements detected. Saliently, iron is low in both (~0.6%). This suggests that the color is of organic origin, and therefore of similar chemical composition to the leather itself. High levels of chlorine are related to the high salt content expected from an object found near the Dead Sea.

**Table 4:** EDX analysis of the areas shown in Fig 1^6^ comparing the brown area (S3, blue square) and black area (S2, red square).

Element symbol	Element name	Weight concentration %	
		S3—brown	S2—black
O	Oxygen	34.82	39.06
C	Carbon	30.74	34.64
N	Nitrogen	14.12	11.86
Ca	Calcium	11.98	8.77
Cl	Chlorine	6.22	3.66
Mg	Magnesium	0.77	0.69
Fe	Iron	0.66	0.59
Na	Sodium	0.34	0.28
S	Sulfur	0.21	0.13
Al	Aluminum	0.07	0.13
Si	Silicon	0.07	0.21

**Fig 17:**
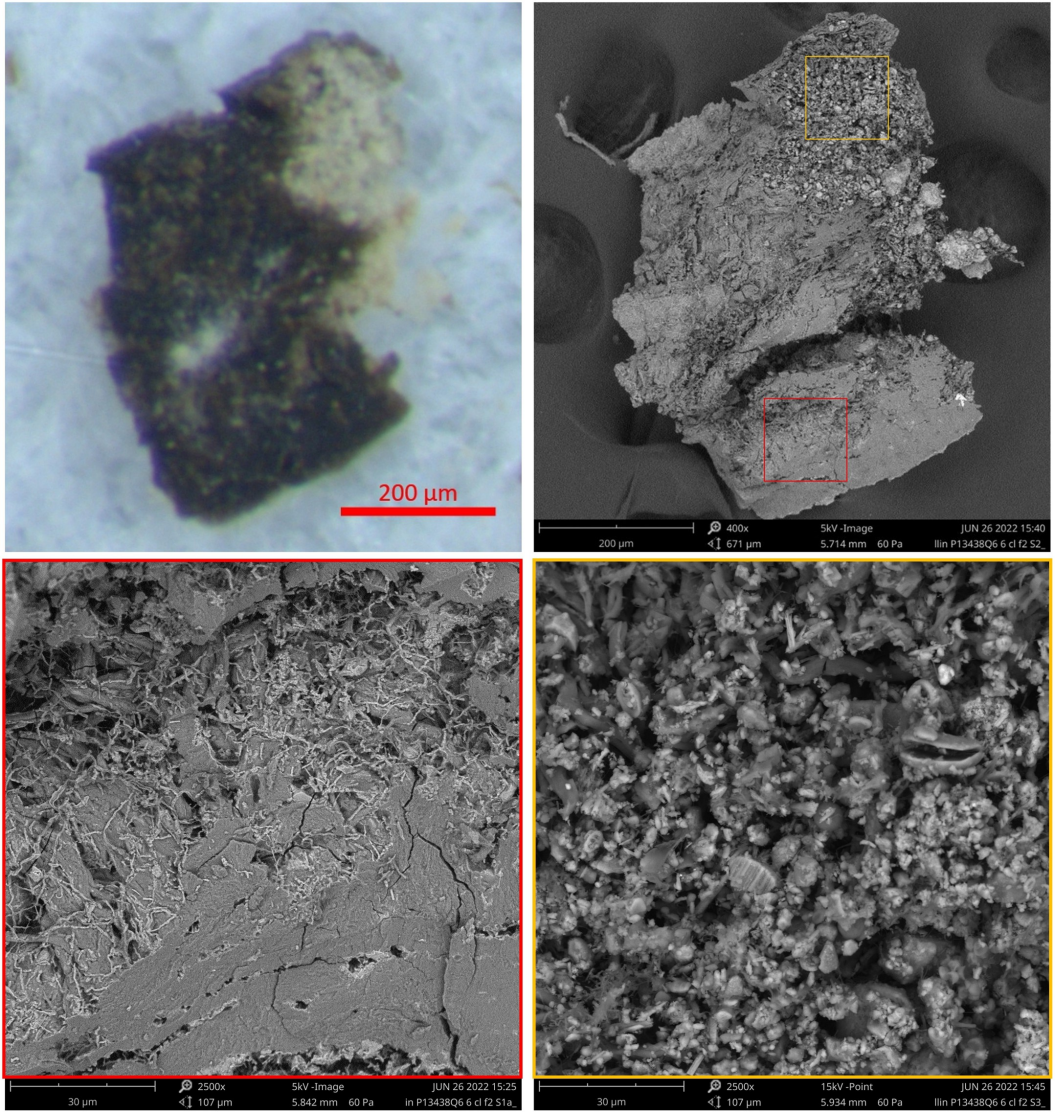
Fragment 2: Photomicrograph and SEM-BSD images. Upper left: Photomicrograph of Fragment 2. Upper right: SEM-BSD image with red square marking black area and orange square marking light colored, mineral coated area. Lower left: SEM-BSD image of red square magnified 2500×. Lower right: SEM-BSD image of orange square magnified 2500×. (Photos: Yonah Maor).

Similar results were obtained from the SEM examination of Fragment 2. Fig 17 shows that the mostly black fragment is gelatinized across most of the surface, with very little fiber structure visible except for the small, calcified fibrils. Zooming in on the mineralized area shows a mix of different materials (based on crystal structure, size and composition). The averaged EDX analysis showed the mineral area included elevated levels of silicon and aluminum, as expected for most soils.

## Discussion

In the thirteen tefillin cases we analyzed using multispectral imaging in the near infrared wavelength 924 nm, no traces of carbon-based pigments were found. Additionally, in the Raman spectroscopy conducted on the Qumran Cave 11 inked parchment fragment (Box 1010), the dominant peaks were amorphous carbon that originate from soot (the pigment in carbon-based ink), whereas in fragments from tefillin case no. 1, no amorphous carbon peaks were identified. These results all suggest that the very dark to black surfaces on the analyzed tefillin cases were not the result of the application of a carbon-based paint.

The Raman analysis also did not detect any trace of an iron-based dye on fragments from tefillin case no. 1. FTIR spectroscopy conducted on fragments from this case likewise revealed no sign of iron-based dying, which we could have expected to see as increased absorbance at 1508 cm^-1^ as seen in iron-gall ink [42]. Similarly, SEM-EDX analyses of fragments from this tefillin case show low concentrations of iron in comparable levels in both brown and black areas. In iron-based ink or colorant, iron is the main element [43–45] and is in higher concentration compared to lighter colored areas [46]. These results suggest that iron-gall pigments were not used in coloring these samples black.

No indications of other organic compounds beyond the leather (collagen and tannins) were observed in the FTIR spectroscopy conducted on fragments from tefillin case no. 1. This suggests that no significant amount of organic binders, such as gum arabic, are present.

*All the above suggests that our originally posited hypothesis—namely*, *that the tefillin cases were colored black using either carbon paint or iron-gall paint or dye—is unlikely*.

In light of these conclusions, a more likely hypothesis is that the very dark to black color on the surfaces of all seventeen tefillin cases is the result of natural degradation of the leather—specifically gelatinization and melanoidin formation. Most studies on melanoidin pigments come from the food industry as they typically form during cooking [47]; however, they also form slowly at room temperature [48] and in our bodies, as part of the aging of collagen, for example in eyes and skin [49, 50]. They have also been indicated as the cause of browning in parchment [51] and in the Dead Sea Scrolls in particular [39, 41].

FTIR analysis ruled out the likelihood that oxidation is a significant cause of the dark colors on analyzed fragments from tefillin case no. 1. SEM examination of these fragments, on the other hand, indicated complete gelatinization in the black areas. Because gelatinization is expected in parts of the skin which have had the most interaction with the environment, it is not surprising that the grain surfaces of the tefillin cases would be the most affected by gelatinization—as these would have come into contact with the sweat and oils on the skin and hair of tefillin practitioners during use and with environmental moisture in the caves post-deposition. We hypothesize that gelatinization accounts for the very dark to black color also on the tefillin cases which in the present study we were unable to analyze with SEM, including the tefillin case studied by Ronald Reed whose black surface he assumed to have been caused by “a black dyestuff used for colouring this leather article” [15 p. 299] as noted above. This hypothesis is consistent with the discrepant depth of penetration of dark color found on sections created by different insect holes in tefillin case no. 1 (noted above); the holes with deeper penetration were likely made very early on, while the others which do not show the interior darkening were likely created more recently in terms of the item’s lifespan.

Although it is hypothetically possible that darkening through gelatinization could have been induced intentionally by ancient tefillin manufacturers, this seems unlikely as the extent of degradation required to produce a dark brown or black color would also alter the physical properties of the leather drastically, making it hard and brittle. In addition, the observed sporadic pattern of dark and light areas is more consistent with natural damage in a cave that is mostly dry but can have water dripping or pooling occasionally. This should be confirmed in future studies by careful examination of the full tefillin case under high magnification to observe the distribution of blackened areas.

The fact that at least some tefillin cases were *not* intentionally colored black is most evident on the three tefillin cases which display a brown color (tefillin cases nos. 18, 19, 20 in Table 2). These tefillin cases were probably exposed to post-depositional effects somewhat different (i.e., drier) from those to which the seventeen black tefillin cases had been exposed.

Our suggestion that none of the tefillin cases or straps analyzed here had been intentionally colored black in antiquity raises the question why the ancient Jews who manufactured these artifacts and put them to ritual use would have disregarded the regulation found in rabbinic literature which prescribes that tefillin cases and straps must be colored black. Assuming that the corpus of tefillin cases from the Judean Desert is generally representative of practices which were widespread among Jews living in the first and early second centuries CE, it seems reasonable to suggest that the Judean Desert tefillin represent practices during a period of time *prior* to when the rabbinic prescription on this matter was widely practiced—or perhaps even conceived. Although the rule that tefillin cases and straps must be black is said to be a “law given to Moses at Sinai”, this assertion should hardly be taken as some sort of historical claim of an extraordinarily ancient practice. Indeed, the Babylonian Talmud cites alternative traditions which would allow leather straps colored green or white (traditions which remain significant despite the fact that the later editorial stratum of the Talmud harmonized these traditions with R. Isaac’s dictum) [6]. Instead, Christine Hayes has described the attribution of this rabbinic rule to “Moses from Sinai” as “an effort to legitimize rabbinic practices regarding tefillin in the face of deviant and sectarian practices” [52 p. 95]. Our present study suggests that the kind of non-blackened tefillin which the Amoraic rabbis rejected as “deviant” in their own times may well have been quite common in earlier times.
